# CO_2_-Based Encapsulation of Rutin-Rich Extracts from Black Elderberry Waste Using Advanced PGSS Process

**DOI:** 10.3390/foods13233929

**Published:** 2024-12-05

**Authors:** Zorana Mutavski, Senka Vidović, Rita Ambrus, Katarina Šavikin, João Baixinho, Naiara Fernández, Nataša Nastić

**Affiliations:** 1 Faculty of Technology, University of Novi Sad, Boulevard cara Lazara 1, 21000 Novi Sad, Serbia; zmutavski@mocbilja.rs (Z.M.); senka.vidovic@uns.ac.rs (S.V.); 2 Institute for Medicinal Plants Research Dr. Josif Pančić, Tadeuša Košćuška 1, 11000 Belgrade, Serbia; ksavikin@mocbilja.rs; 3 Faculty of Pharmacy, Interdisciplinary Excellence Centre, Institute of Pharmaceutical Technology and Regulatory Affairs, University of Szeged, Eötvös Street 6, H-6720 Szeged, Hungary; ambrus.rita@szte.hu; 4 Instituto de Tecnologia Química e Biológica António Xavier, Universidade Nova de Lisboa, Av. da República, 2780-157 Oeiras, Portugal; jbaixinho@ibet.pt; 5 iBET, Instituto de Biologia Experimental e Tecnológica, Apartado 12, 2781-901 Oeiras, Portugal; naiara.fernandez@ibet.pt

**Keywords:** encapsulation, black elderberry waste, rutin preservation, particles from gas-saturated solutions (PGSS), amphiphilic carriers

## Abstract

For the first time, rutin-rich extracts from black elderberry waste (BEW) were encapsulated using the particles from gas-saturated solutions (PGSS) method to improve the preservation of rutin. The extracts used in this study were obtained using five different extraction techniques under optimal conditions, as follows: conventional solid–liquid extraction (SLE) and four non-conventional techniques—ultrasound-assisted extraction (UAE), microwave-assisted extraction (MAE), enhanced solvent extraction (ESE), and supercritical CO_2_ pretreatment—followed by ESE (SFE-CO_2_ + ESE). The PGSS process of the obtained extracts was performed using two amphiphilic carriers, glycerol monostearate (GlyMS) and gelucire (Gel), in a mass ratio of 1:6, in favor of the carrier. The efficiency of the PGSS process was evaluated based on the encapsulation yield (EnY), encapsulation efficiency (EE), and physical properties of the encapsulated extracts. The results showed that the SLE extract encapsulated with GlyMS had the highest EnY (92.47%). The Gel only in combination with the ESE extract exceeded the 50% efficacy threshold, with an EnY of 55.18%. The encapsulated SLE extract with Gel showed excellent flow properties and the highest EE (98.91%). These results emphasize the advantages of the PGSS process, including its efficiency and adaptability to produce encapsulated rutin-enriched BEW extracts for pharmaceutical, nutraceutical, and cosmetic applications.

## 1. Introduction

About 80% of the world’s population in developing countries relies on herbal medicines as their main source of healthcare [1]. Deeply rooted in plants and traditional knowledge, these natural remedies have been essential in local healing practices for generations. Due to their accessibility, affordability, and cultural significance, herbal medicines are often the first defense against various diseases. Rutin, a sought-after natural flavonoid found in various vegetables and fruits, is an important compound in over 130 approved herbal medicines worldwide [2,3]. Rutin, also known as vitamin P, is a flavonol glycoside in which the phenolic segment of quercetin is tightly bound to rutinose—a sugar that improves the water solubility of the molecule. Through enzymatic hydrolysis by glucosidase, rutin is converted into two compounds: the flavonol quercetin and the disaccharide rutinose. The intestinal microflora can absorb these compounds [4].

In recent years, numerous studies have investigated the various biological effects of rutin and have identified a broad spectrum of positive properties. These include its known antioxidant abilities [5], antimicrobial abilities [6,7], potential antithrombotic effects [8], cardioprotective properties [9], as well as neuroprotective activities [10]. Positive effects on liver health, function, and integrity have also been observed [11]. Among the many effects of rutin, its anticarcinogenic properties [12,13,14] and its potential as an antidiabetic agent [15,16] are the most studied and highlighted. These significant findings emphasize the versatile therapeutic potential of rutin and contribute to the growing body of evidence for its role in promoting health and combating various diseases.

With the growing demand for rutin isolated from natural resources, it is important to investigate rutin-rich plants and extraction methods for rutin recovery. Numerous extraction techniques have been investigated to extract rutin from diverse plant materials quantitatively. These techniques include traditional solid–liquid extraction (SLE) [17,18], subcritical water extraction (SWE) [19,20], microwave-assisted extraction (MAE) [21], ultrasound-assisted extraction (UAE) [22], supercritical fluid extraction (SFE) [23], and solid-phase microextraction (SPM) [24].

Nowadays, the utilization of agri-food waste and by-products has gained significance as a research topic to improve the sustainability of food resources. In our previous research, SLE, UAE, and MAE were employed to extract bioactive compounds from black elderberry waste (BEW), resulting in substantial concentrations of isolated rutin [25]. Additionally, enhanced solvent extraction (ESE), with and without SFE-CO_2_ pretreatment, was also investigated to determine the optimal parameters for the extraction of bioactive compounds from BEW [26]. This waste, which consists of the peel and seeds left after juice production, has been recognized as a valuable source of nutrients and bioactive secondary metabolites, including anthocyanins (cyanidin 3-O-sambubioside and cyanidin 3-O-glucoside) and flavonoids, with rutin being the most abundant [27,28]. In general, the main disadvantages of liquid plant extracts are the degradation of high-value bioactive compounds over time and reduced bioavailability. One strategy to mitigate these issues is to microencapsulate these extracts in carriers through microencapsulation methods. Various techniques to enhance the therapeutic potential and improve the absorption of functional foods within the digestive system have been explored [29,30,31]. Specifically, focusing on bioactive compounds from natural sources, the goal is to preserve and convert them into more stable forms, thereby extending their shelf life and increasing their bioavailability. The particles from gas-saturated solutions (PGSS) technology stands out as a high-pressure CO_2_-based encapsulation method capable of producing powders with superior stability properties [32,33]. This process offers several advantages, including a short processing time, the use of low temperatures, and the creation of uniform powders.

To our knowledge, no previous studies specifically address the encapsulation of rutin from BEW extracts in amphiphilic carriers using the PGSS process. The extracts used in this study were obtained by SLE, UAE, SWE, ESE, and SFE-CO_2_ + ESE. Such encapsulated powders could be potentially incorporated into functional food formulations, providing a stable delivery system for bioactive compounds like rutin, which is valued for its health-promoting properties. Therefore, the main objective of this study was to compare the suitability of two carriers, glycerol monostearate (GlyMS) and gelucire (Gel), in the PGSS encapsulation process of rutin-rich extracts. These carriers were selected due to their food suitability and their approval as food additives in the EU. The physical and morphological properties of the resulting encapsulations were analyzed. These properties include encapsulation yield (EnY), encapsulation efficiency (EE), particle size, bulk, and tapped density, as well as parameters that are important for evaluating the flow properties of the powder, such as the Carr index and the Hausner ratio.

## 2. Materials and Methods

### 2.1. Chemicals

Dimethyl sulfoxide (DMSO) with a purity of 99.95% (Carlo Erba Reagents, Val de Reuil, France) was used as an agent for recovering rutin from powders. HPLC-grade acetonitrile (Merck, Darmstadt, Germany) and ultrapure water prepared with a Milli-Q purification system (Merch Millipore, Guyancourt, France) were employed. The rutin standard with a purity of more than 96% (Extrasynthese, Genay, France) was used as a standard. Imwitor^®^ 600 (Sasol, Witten, Germany) and GlyMS and Gel (Gattefosse SAS, Saint-Priest, France) were used for encapsulation purpose. For the PGSS experiences, pure-grade CO_2_ was used (99.95%, Air Liquide, Algés, Portugal). All other chemicals meeting analytical-grade standards were used without further purification.

### 2.2. Sample Material and Preparation of BEW Extracts

BEW (*Sambucus nigra* L.) was sourced from NISHA d.o.o., a company in Belgrade (Serbia) specializing in the harvesting and processing of forest fruits. The raw material underwent a process that included drying in a vacuum dryer and grinding in a mixer. For the extraction of rutin, the optimal extraction parameters determined in our previous studies were used [25,26]. For SLE, the optimal conditions were 30% EtOH as a solvent and an extraction time of 24 h. For UAE, the same solvent was used with an ultrasound treatment of 7 min at an amplitude of 60%. SWE extraction parameters were set to a temperature of 120 °C, with an extraction time of 20 min and a pressure of 20 bar. The extraction ratio of BEW to solvent was kept constant at 1:10 for SLE, UAE, and SWE. For ESE, two conditions were tested, as follows: with and without pretreatment with supercritical CO_2_ (SFE-CO_2_) at a pressure of 200 and 350 bar, respectively. The solvent for these extractions was a CO_2_:EtOH:H_2_O mixture in a ratio of 80:1:19. After extraction, the solvents were evaporated under vacuum conditions, and the resulting extracts were prepared for subsequent analysis.

### 2.3. Particles from Gas-Saturated Solutions (PGSS)

The BEW extracts were encapsulated using the PGSS^®^ technique in two carriers, GlyMS and Gel, using water and an emulsifier. Table 1 shows the nomenclature used for the powders obtained, distinguishing between PGSS 1–5, obtained with GlyMS, and PGSS 6–10, obtained with Gel.

The encapsulation process was carried out in a precisely controlled environment using a thermostatically controlled high-pressure stirring vessel (PGSS^®^ Separex Supercritical and High-Pressure Technology) maintained at 70 °C. To a mixture of 3.5 g BEW and the selected carrier, 1 mL water and 0.1 mL emulsifier (Imwitor^®^ 600) were added. The mass ratio of BEW to carrier was fixed at 1:6. Carbon dioxide was introduced into a 50 cm^3^ electrically thermostated stirring vessel containing the dispersion using a high-pressure piston pump (29723-71, Haskel International Inc., Burbank, CA, USA) until the desired working pressure (120 bar) was reached. After a 15-minute equilibrium time, stirring at 150 rpm, the pressure in the mixture was released through an automatic pressure release valve. It was then atomized through a 710 μm diameter two-substance nozzle with external mixing (Spraying Systems Co., Air atomization 1/4J-SS, Separex, Champigneulles, France) into a cyclone, where it was mixed with compressed air at 7 bar for better drying. The resulting particles were collected under atmospheric pressure in an 18 L collection container.

### 2.4. Powder Characterization

#### 2.4.1. Encapsulation Yield (EnY)

The EnY (Equation (1)) was determined by calculating the ratio between the actual mass of the dry powder (m_p_) collected from the device’s receiving vessel and the mathematically derived expected powder mass (consisting of the mass of the dry residue of the collected extract and the carrier mass, m_ep_), as follows:

(1) $$EnY\;\left(\%\right)=\frac{mp}{mep}\times 100$$

#### 2.4.2. HPLC Analysis and Encapsulation Efficiency of Rutin

Rutin was analyzed using an Agilent 1200 RR system (Agilent, Waldbronn, Germany) equipped with a diode array detector. A reversed-phase Lichtrospher RP-18 (Agilent, Waldbronn, Germany) column (250 mm × 4 mm, 5 μm) was used, and the column temperature was maintained at 25 °C. The mobile phase consisted of solvent A (10%, *v*/*v* solution of formic acid in water) and solvent B (acetonitrile), using the following gradient elution profile: 1% B 0–0.5 min; 1–7% B 0.5–1 min; 7% B 1–4 min; 7–10% B 4–7.5 min; 10–14% B 7.5–11.5 min; 14–25% B 11.5–15.5 min; 25–40% B 15.5–18.5 min; 40–75% B 18.5–22 min; and 75% B 22–25 min. The injection volume was 10 μL, the flow rate was 1 mL/min, and detection was performed at wavelengths of 290, 350, and 520 nm. The rutin content was determined using a calibration curve, and the results are expressed in milligrams per gram of dry weight (mg/g powder). The correlation coefficient of the calibration curve was 0.9999, and the target compound showed good linearity in the range of 50 to 500 µg/mL. The encapsulation efficiency (EE) was determined based on the proportion of quantified and expected rutin content in powders (%). The expected concentration of rutin is the theoretical amount of rutin present in powders, considering the dilution that occurs when carriers are added in an extract-to-carrier ratio of 1:6.

#### 2.4.3. Bulk and Tapped Densities, Carr Index, and Hausner Ratio

For the determination of densities, the samples were poured into 5 mL graduated cylinders, and the values for bulk density (ρ_bulk_) were derived from the mass/volume ratios. For the tapped density (ρ_tap_), a STAV 2003 Stampf volumeter (Engelsmann A.G., Luwigshafen, Germany) was used as the tapping device, and the results were the mass/tapped volume ratios. A method for measuring the bulk density and tapped density of powders is described in Chapter 2.9.34 of the European Pharmacopeia (2010) [34]. The flowability and cohesion values of the powders were determined in terms of the Carr index (CI) and the Hausner ratio (HR), respectively, which were calculated using Equations (2) and (3), as follows:

(2) $$CI=\frac{\left(\rho tap-\rho bulk\right)}{\rho tap}\times 100$$

(3) $$HR=\frac{\rho tap}{\rho bulk}$$

#### 2.4.4. Scanning Electron Microscopy (SEM) and Particle Size

The morphology of the particles in the obtained powders was analyzed by SEM at 10 kV using a Hitachi S4700 instrument from Hitachi Scientific Ltd. of Tokyo, Japan. The particle diameter distribution of the samples was determined by SEM analysis.

For the microparticles, the particle size was determined using scanning electron-microscope pictures with Image J Software 1.53t.

### 2.5. Statistical Analysis

Analyses were performed in triplicate, and the results are expressed as means ± standard deviations (SD). The significance between the means was determined at a confidence level of *p* < 0.05. A one-way ANOVA was employed for the statistical analysis, supplemented by Tukey’s test for further comparison. The obtained data were statistically analyzed using TIBCO Software Inc., version 14.0.0.15 (Palo Alto, CA, USA).

## 3. Results and Discussion

### 3.1. BEW Powder Characterization

#### 3.1.1. Encapsulation Yield

The encapsulation yield (EnY) serves as a decisive parameter that reflects the economic feasibility of the encapsulation process. If the EnY value falls below the 50% threshold, the physical parameters of the encapsulation process must be re-evaluated. Alternatively, a carrier can be added to the feed mixture to increase the EnY value. If none of these measures prove to be effective, a change in the encapsulation technology may be justified [35,36]. Typically, the EnY percentage is below 100%, due to factors such as powder adherence to the device walls. This can be due to various reasons, including electrification of the particles and unfavorable encapsulation conditions, leading to increased moisture of the powder. In the PGSS process, the adhesion of the particles to the device can be caused by the increased pressure during depressurization. At this point, the powder dries under high pressure and bounces against the cyclone walls, causing it to stick. In the field of microencapsulation, the PGSS process is suitable for water-soluble active compounds and carriers with different polarities.

In the present study, in the first step of the PGSS process, the evaporated extracts were mixed with the carriers in a stirred vessel with the addition of an emulsifier. Since five different extraction techniques were used for the extraction of bioactive compounds from BEW, it was expected that the extracts prepared for encapsulation would have different chemical compositions in terms of polarity. Consequently, an appropriate amount of emulsifier should be used to ensure the formation of a consistent mixture between the dry BEW extract and the amphiphilic carrier to enable efficient homogenization and encapsulation. In the research conducted by Nastić et al. [31], GlyMS has proven its effectiveness as a suitable carrier for the encapsulation of ethanolic extracts from black raspberry waste using the PGSS method. Similarly, Mudrić et al. [37] achieved success utilizing Gel, supplemented with an emulsifier, to produce microparticles from hydrophilic extracts derived from gentian roots through freeze drying.

**Figure 1:**
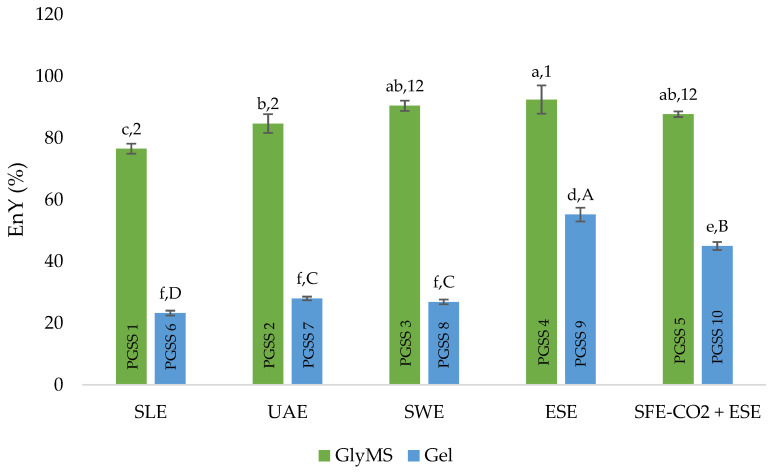
EnY of BEW powders obtained from five different extraction techniques by PGSS with GlyMS and Gel. The values of all samples were compared. Different lowercase letters above the bars signify statistically significant differences between samples at the *p* < 0.05 level. Different numbers above the bars signify statistically significant differences between samples with the GlyMS carrier at the *p* < 0.05 level. Different uppercase letters above the bars signify statistically significant differences between samples with the Gel carrier at the *p* < 0.05 level.

According to the results shown in Figure 1, the powders produced using GlyMS had higher EnY values (76.52−92.47%), regardless of the type of extraction.

In particular, the ESE (PGSS 4) showed the highest EnY with GlyMS at 92.47%, indicating a strong compatibility between this extract and GlyMS. On the other hand, the efficiency of the Gel as a carrier varied between the different extracts. While the Gel with the ESE extract achieved the highest EnY value of 55.18%, it generally proved to be less effective than GlyMS when encapsulating BEW extracts. This could be due to problems during the depressurization step of the PGSS process, possibly related to the adhesion of the material to cell walls and tubes. The SLE extract consistently provided the lowest EnY value with both carriers, as observed in PGSS 1 and PGSS 6. In the study by Nastić et al. [31], it was shown that the EnY of raspberry pomace extracts obtained with the PGSS technique was also lower for SLE extracts compared to UAE extracts of the same raw material with the GlyMS carrier. A pressure of 100 bar was applied, as well as an extract-to-carrier ratio of 1:6. Remarkably, the EnY for the SLE extracts was 51.03%, signifying a lower value compared to the corresponding UAE extracts, which demonstrated a superior EnY of 64.47% [31]. In food and pharmaceutical applications, GlyMS is often used as an anti-caking agent to prevent the formation of lumps or clumps in powdered or granulated products [38]. This use may be responsible for a higher EnY compared to that of Gel powders, whose particles were more likely to adhere to the surfaces of processing equipment. There was no observed correlation between extraction yields of the extracts [25,26] and EnY of the obtained powders with both carriers.

#### 3.1.2. Encapsulation Efficiency

To our knowledge, the encapsulation of rutin-rich hydrophilic BEW extracts by the PGSS process and the quantification of rutin from the resulting powders have not been previously studied. In a notable study, Gonçalves et al. [39] successfully encapsulated pure quercetin using PGSS technology under specific conditions, including a pressure range of 76.8–117.7 bar, a pre-expansion temperature between 109.4 and 132.5 °C, and a spray tower temperature ranging from 64.7 to 75.1 °C. The chosen carriers for this process were Pluronic^®^ L-121 and soy lecithin.

The determination of rutin content in the obtained PGSS powders in the present research was performed using HPLC-DAD analysis to assess the extent to which the structure of rutin was preserved throughout the process. The rutin content in the powders ranged from 288.27 to 655.73 µg/g powder (Table 2).

The PGSS 7 powder exhibited the highest rutin content (655.73 µg/g powder), while the lowest value (288.27 µg/g powder) was observed in PGSS 3. PGSS 6 (SLE) showed a high rutin content (589.22 µg/g powder) and exceptional EE (98.91%). In contrast, the extraction methods with subcritical water (PGSS 3 and PGSS 8) showed the lowest EE (39.22% with GlyMS and 42.10% with Gel). The PGSS 2 sample was selected as the optimal one, as it has the highest rutin concentration compared to the powders with GlyMS. It also has a threefold higher EnY (84.69%) compared to PGSS 6 (28.01%), which had the highest rutin concentration compared to all other powders. The high rutin content and encapsulation efficiency of these powders make them suitable candidates for incorporation into functional food products, where the potent antioxidant properties of rutin could contribute to improved health outcomes and enhance the nutritional profile of the final products.

When the EE was calculated for PGSS 4, PGSS 5, PGSS 9, and PGSS 10, the values were above 100%, indicating that the conditions selected for the PGSS formulation were not suitable for those extracts. An encapsulation efficiency (EE) higher than 100% in the context of PGSS with supercritical CO_2_ typically indicates a physical phenomenon. Upon rapid depressurization, a larger amount of the active compound could precipitate rapidly, forming particles with low carrier content. The distribution of the active compound might not be uniform within the particles. Certain regions might have a higher concentration of the active compound due to localized supersaturation. During the encapsulation process, particularly under high pressure and temperature, the particles may aggregate or coalesce. This could lead to capturing more active principles than initially expected.

Supercritical CO_2_ can cause a supersaturation effect, where the solubility of the active compound in the CO_2_ phase is extremely high. ESE extracts and SFE-CO_2_ + ESE extracts, due to their extraction process and composition, present low affinity for CO_2_. Compounds with low affinity for CO_2_ may have poor solubility in the supercritical CO_2_ phase, making it difficult to dissolve these compounds into the encapsulating matrix. Low solubility can lead to inefficient loading of the active compound into the matrix. The compound might precipitate out of the CO_2_ phase before it can be properly encapsulated, leading to agglomeration and poor particle formation, increasing the errors in EE calculations.

There is a study looking at the encapsulation of rutin by freeze drying and oven drying using different coating materials, such as starch, egg albumin, and lipid carriers [40]. The authors presented research highlighting the benefits of lipid carriers in improving the bioavailability of rutin in oral dosage forms and increasing EE by up to 80%. In a study by Gali et al. [41], rutin extracted from *Ruta chalepensis* at a concentration of 200.6 mg/g dry extract was encapsulated with a zein/gum arabic mixture prepared by antisolvent precipitation. An analysis using the HPLC method revealed that the resulting powder contained 30.1% of the original rutin content, which was up to three times lower than that observed in our study.

### 3.2. Physical Characteristics of Powders

#### 3.2.1. Bulk and Tapped Densities, Carr Index, and Hausner Ratio of BEW Powder

The bulk and tapped densities of powders are crucial parameters with significant implications for manufacturing processes, dosage formulation in pharmaceuticals, storage, transportation, powder flowability, quality control, and formulation optimization. These density measurements provide essential insights into powder characteristics, guiding the design of efficient production systems and ensuring consistent product quality across various industries [42,43]. A high bulk density improves powder uniformity by minimizing the air space between the particles, thereby increasing the overall stability. This property plays a crucial role in ensuring the quality and reliability of the final products. From this perspective, the PGSS 5 powder exhibited the highest bulk and tapped density among the powders with GlyMS. Conversely, the highest bulk and tap densities were observed within the Gel powders for PGSS 7 and PGSS 8. Comprehensive results are summarized in Table 3 and provide valuable insights into the specific properties of the powders studied.

The bulk density of powders is influenced by factors such as particle size, shape, porosity, moisture content, compressibility, flowability, consolidation history, and external forces during measurement. These factors collectively determine how closely particles can pack together, affecting the overall bulk density of the powder. The low bulk density of some powders could be due to the initial solution’s higher viscosity, leading to larger particle sizes [44]. The bulk and tapped densities are crucial for calculating the CI and HR, which indicate powder compressibility and flowability. These parameters provide valuable insights into powders’ handling and processing characteristics, guiding quality control and process optimization in diverse industries. A CI of less than 15 means that microparticles with good flow properties were obtained [45]. In contrast, a CI greater than 25 indicates poor flowability. In the present study, the PGSS 6 microparticles had the lowest CI of 14.93 (PGSS 6) and showed the best flow properties (good flowability) compared to the other samples. The different flow properties of these powders cannot be explained by a specific or exact design. Ultimately, the importance of their flowability and cohesiveness depends on the desired future use of the powder, and the choice of a carrier depends on the desired shape and application of the final product. To enhance the flowability and cohesiveness of powders, we should consider optimizing particle size distribution, employing suitable surface treatments, managing moisture content, and utilizing granulation techniques. While adding carriers can improve the flow by facilitating better dispersion, their impact on cohesiveness depends on the specific properties of the carrier and its compatibility with the powder material. Careful selection and testing of carriers are essential to achieve the desired balance between improved flow and controlled cohesion in powder formulations.

#### 3.2.2. SEM Analysis and Particle Size of BEW Powder

**Figure 2:**
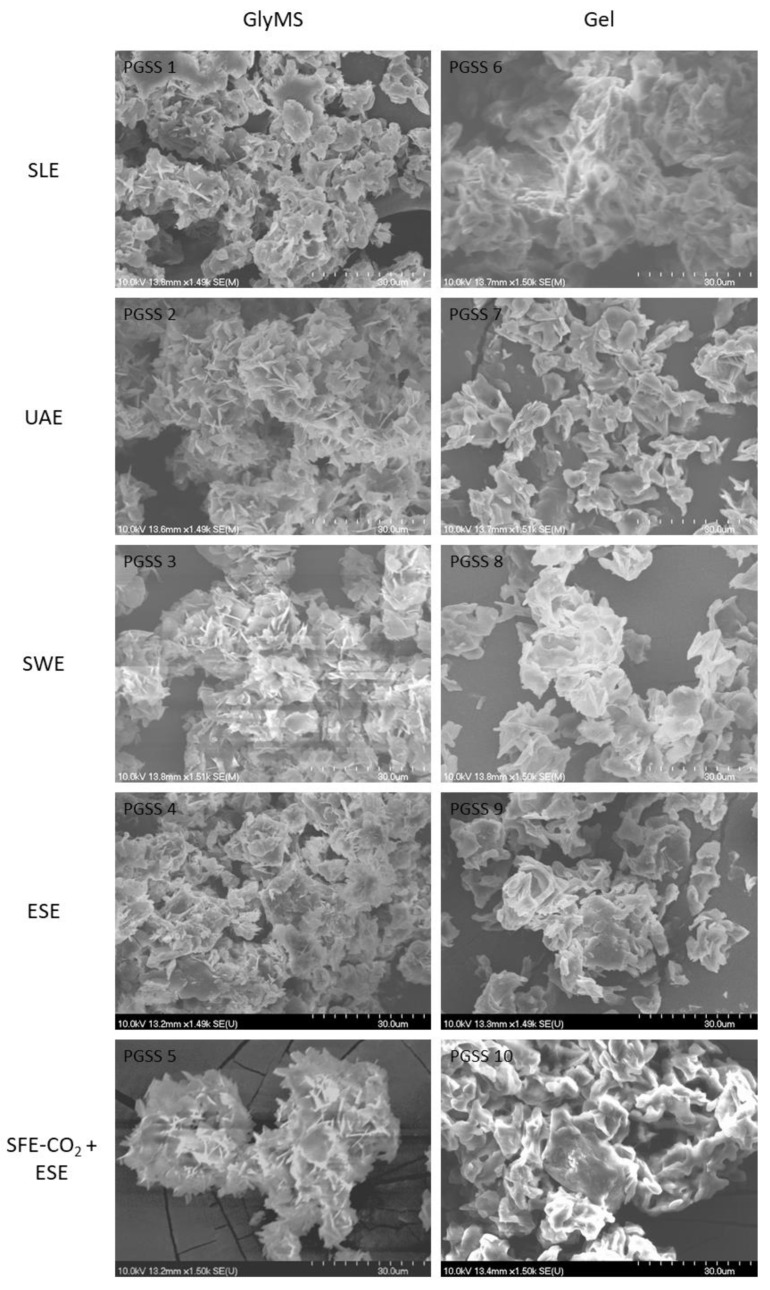
SEM analysis of BEW microparticles.

The morphological examination of encapsulated BEW extracts within an amphiphilic carrier was conducted through SEM analysis, as illustrated in Figure 2.

Notably, the resulting powders exhibited a surface consistency similar to that of flakes, as evidenced by the morphological characteristics observed. In Figure 2, it is apparent that particle aggregation is present in all combinations of extracts and carriers. Particularly noteworthy is the observation that microparticles containing Gel as a carrier displayed more rounded edges compared to those with GlyMS. The formation of crystal-like particles can likely be attributed to the operating pressure, where dry microparticles collide with cyclone walls upon pressure release. This phenomenon aligns with the findings of Klettenhammer et al. [46], who reported similar behavior in the microencapsulation of linseed oil enriched with extracts from carrot pomace using the PGSS process. Their study also noted the presence of non-spherical particles and observed an increase in aggregation with higher working pressures. These insights contribute to our understanding of the morphological aspects of encapsulated extracts and offer parallels with related research in the field. The particles show a surface consistency similar to that of flakes and flower-like structures (desert roses).

The particle sizes of the BEW microparticles, as measured with SEM images, are listed in Table 3. No interpretable differences were found in the sizes of the microparticles containing Gel and GlyMS as carriers. Solvent extraction with SFE-CO_2_ pretreatment resulted in the largest particle diameters with both carriers, with a wide particle size distribution. This is possible due to strong aggregation, which may happen due to the addition of water to a carrier material [32]; moreover, it is difficult to identify individual particles in the sample. Although all sample sizes showed a wide distribution, PGSS 3 and PGSS 7 proved to be the greatest, and the standard deviation of PGSS 8 was also below 6 µm. The sample’s flow values were acceptable, probably due to the more homogeneous distribution compared to that of the other samples. The optimal particle size for increasing the bioavailability of dry extract powders generally falls in the range of 1 to 10 µm. This size range enhances dissolution rates and absorption in the gastrointestinal tract, improving bioavailability. However, balancing particle size reduction while maintaining stability is crucial for effective bioavailability enhancement [47,48].

## 4. Conclusions

BEW extracts obtained by different methods—SLE, UAE, SWE, ESE, and SFE-CO_2_ + ESE—were encapsulated with PGSS and two carriers, GlyMS and Gel. The EnY for PGSS with GlyMS was remarkably high, reaching a peak value of 92.47% for PGSS 4 (ESE) powder. In contrast, the EnY values with Gel as a carrier were significantly lower and ranged from 23.27% to 55.18%. Nevertheless, the Gel-encapsulated extracts exhibited better flow properties, including bulk density, tap density, CI, and HR. PGSS 2 in the GlyMS carrier group and PGSS 6 in the Gel carrier group contained the highest concentrations of rutin at 540.37 and 655.73 µg/g powder, respectively. This shows that both GlyMS and Gel are suitable for encapsulation, depending on the desired properties of the final product. PGSS 2 was considered optimal due to its significant EnY (84.69%) and concentration of rutin. In conclusion, microencapsulation of BEW extracts using GlyMS and Gel as wall materials is a promising approach for the preservation of valuable bioactive compounds, especially rutin, which is known for its potent biological activities and contribution to consumer health. The obtained powders could be incorporated into functional food formulations or dietary supplements to deliver bioactive compounds in a stable and bioavailable form to meet the growing demand for health-promoting foods.

## Figures and Tables

**Table 1:** Nomenclature of powders produced using the PGSS process with two carriers, GlyMS and Gel.

Code	Carrier	Extraction Technique
PGSS 1	GlyMS	SLE
PGSS 2		UAE
PGSS 3		SWE
PGSS 4		ESE
PGSS 5		SFE-CO_2_ + ESE
PGSS 6	Gel	SLE
PGSS 7		UAE
PGSS 8		SWE
PGSS 9		ESE
PGSS 10		SFE-CO_2_ + ESE

Abbreviations: PGSS—particles from gas-saturated solutions; GlyMS—glycerol monostearate; Gel—gelucire; SLE—solid/liquid extraction; UAE—ultrasound-assisted extraction; SWE—supercritical water extraction; ESE—enhanced solvent extraction; SFE-CO_2_ + ESE—supercritical CO_2_ pretreatment in combination with enhanced solvent extraction.

**Table 2:** Concentrations of rutin in BEW powders (µg/g powder) obtained from three different extracts encapsulated by PGSS with GlyMS and Gel.

	C Rutin (μg/g dry Extract)	C rutin Expected (μg/g Powder)	C rutin in PGSS 1–3 (μg/g Powder) */***	C rutin in PGSS 6–8 (μg/g Powder) **/***
SLE	4170.00	595.71	362.87 ± 10.32 ^b,4^ (EE 60.91%)	589.22 ± 13.72 ^B,2^ (EE 98.91%)
UAE	8860.00	1265.71	540.37 ± 14.24 ^a,3^ (EE 42.69%)	655.73 ± 10.81 ^A,1^ (EE 51.81%)
SWE	5144.42	734.92	288.27 ± 7.66 ^c,5^ (EE 39.22%)	309.44 ± 3.22 ^C,5^ (EE 42.10%)

* Different lowercase letters above the column signify statistically significant differences between powders with glycerol monostearate (GlyMS) carrier at the *p* < 0.05 level. ** Different uppercase letters above the column signify statistically significant differences between powders with gelucire (Gel) carrier at the *p* < 0.05 level. *** Different numbers above the columns signify statistically significant differences between all powders at the *p* < 0.05 level. Abbreviations: C—concentration; EE—encapsulation efficiency; GlyMS—glycerol monostearate; Gel—gelucire; SLE—solid/liquid extraction; UAE—ultrasound-assisted extraction; SWE—supercritical water extraction.

**Table 3:** Physical characteristics and flowability of the obtained BEW microparticles.

Code	Bulk Density (mg/mL)	Tapped Density (mg/mL)	Carr Index	Hausner Ratio	Particle Size (µm)	Flow
PGSS 1	70.22 ± 2.12 ^ef^	103.23 ± 5.25 ^d^	31.98 ± 1.42 ^a^	1.47 ± 0.06 ^a^	8.5 ± 7.4 ^b^	Poor
PGSS 2	74.35 ± 2.56 ^de^	100.11 ± 5.85 ^d^	25.73 ± 1.18 ^bc^	1.35 ± 0.03 ^bc^	7.8 ± 8.8 ^b^	Poor
PGSS 3	64.31 ± 2.13 ^f^	84.58 ± 3.36 ^e^	23.96 ± 0.95 ^cd^	1.31 ± 0.03 ^bcd^	6.4 ± 4.5 ^b^	Acceptable
PGSS 4	54.11 ± 2.02 ^g^	73.83 ± 2.54 ^e^	26.71 ± 1.18 ^bc^	1.35 ± 0.03 ^bc^	9.9 ± 6.4 ^b^	Poor
PGSS 5	80.21 ± 2.96 ^cd^	111.27 ± 5.81 ^d^	27.91 ± 0.65 ^b^	1.39 ± 0.03 ^ab^	26.8 ± 22.1 ^a^	Poor
PGSS 6	131.21 ± 4.26 ^b^	154.11 ± 5.85 ^b^	14.86 ± 0.51 ^f^	1.18 ± 0.04 ^e^	11.5 ± 13.9 ^b^	Good
PGSS 7	141.65 ± 4.25 ^a^	172.37 ± 5.36 ^a^	17.82 ± 0.49 ^e^	1.22 ± 0.05 ^de^	7.2 ± 4.3 ^b^	Acceptable
PGSS 8	135.95 ± 7.56 ^ab^	172.84 ± 4.74 ^a^	21.34 ± 0.48 ^d^	1.27 ± 0.04 ^cde^	11.0 ± 5.9 ^b^	Acceptable
PGSS 9	74.65 ± 2.58 ^de^	109.21 ± 5.87 ^d^	31.64 ± 0.77 ^a^	1.47 ± 0.03 ^a^	8.5 ± 7.3 ^b^	Poor
PGSS 10	88.81 ± 3.96 ^c^	129.58 ± 3.84 ^c^	31.46 ± 1.47 ^a^	1.47 ± 0.03 ^a^	13.6 ± 24.8 ^a^	Poor

Distinct letters within a column indicate a statistically significant difference between the samples at a significance level of *p* < 0.05.

## Data Availability

The original contributions presented in this study are included in the article, and further inquiries can be directed to the corresponding author.
